# A Subject-Specific Cerebrovascular CFD Modeling Approach Based on a Multimodal Data-Driven Boundary Calibration Framework: A Proof-of-Concept Study

**DOI:** 10.3390/bioengineering13080861

**Published:** 2026-07-25

**Authors:** Jun Hu, Hongye Li, Xuelian Shen, Yonghao Zhong, Hanxiong Zheng, Yiao Liu, Bin Luo, Jianhang Du

**Affiliations:** 1 Medical Research Center, The Eighth Affiliated Hospital of Sun Yat-sen University, Shenzhen 518033, China; hujun26@mail2.sysu.edu.cn; 2 School of Biomedical Engineering, Sun Yat-sen University, Shenzhen 518107, China; 3 Department of Ultrasound, The Eighth Affiliated Hospital of Sun Yat-sen University, Shenzhen 518033, China; lihy353@mail.sysu.edu.cn (H.L.); shenxlian3@mail.sysu.edu.cn (X.S.); 4 Department of Urology, Shenzhen Futian Second People’s Hospital, Shenzhen 518033, China; zyh136921@163.com (Y.Z.); shmily841677@163.com (H.Z.); 5 Department of Neurosurgery, The Eighth Affiliated Hospital of Sun Yat-sen University, Shenzhen 518033, China; 6 National Health Commission (NHC) Key Laboratory of Assisted Circulation, Sun Yat-sen University, Guangzhou 510080, China

**Keywords:** subject-specific CFD, enhanced external counterpulsation, Circle of Willis, cerebral autoregulation, multimodal physiological monitoring, boundary condition optimization, wall shear stress

## Abstract

Cerebrovascular computational fluid dynamics (CFD) models often rely on generic boundary conditions, which may limit their ability to represent subject-specific hemodynamics and cerebral autoregulation (CA). We propose a multimodal data-driven boundary calibration (MDBC) framework integrating transcranial color-coded Doppler and continuous blood pressure monitoring to optimize individualized outlet resistances. As a proof-of-concept, we evaluated the MDBC framework in a single healthy volunteer at resting baseline and enhanced external counterpulsation (EECP)—a hemodynamic perturbation potentially triggering CA. Compared with conventional open boundary (OB) and static Murray allocation boundary (SMAB) strategies, MDBC achieved closer agreement with in vivo middle cerebral artery (MCA) velocity waveforms under both states. At rest, MDBC’s left MCA relative root mean square error (rRMSE) was 7.19%, versus 22.85% (OB) and 30.89% (SMAB). During EECP, conventional models yielded rRMSEs > 32%, whereas MDBC maintained 11.24%. Meanwhile, MDBC reproduced inter-hemispheric perfusion imbalance, an EECP-induced flow surge in the right MCA, and pronounced wall shear stress increases that were masked by generic boundary strategies. Moreover, MDBC estimated a 25.8% increase in global cerebrovascular resistance during EECP, suggesting the capability of the framework to characterize subject-specific impedance adaptations potentially associated with CA during intervention. These single-subject findings support the technical feasibility of integrating multimodal physiological measurements into cerebrovascular CFD boundary calibration and warrant further validation in larger cohorts and patient populations.

## 1. Introduction

In recent years, computational fluid dynamics (CFD) has been increasingly adopted as a non-invasive tool for investigating cerebrovascular hemodynamics, enabling the high-resolution estimation of intravascular flow fields, pressure distributions, and key biomechanical metrics such as wall shear stress (WSS) [1,2]. However, the reliability of CFD simulations strongly depends on the specification of boundary conditions (BCs) [3], which vary considerably among previous studies. While subject-specific data are occasionally utilized for inlet BCs [4,5,6], outlet BCs are frequently simplified using constant pressure assumptions (e.g., open boundary at 0 Pa) [6,7,8,9] or morphologically driven impedance allocations such as Murray’s law [10,11,12]. Although computationally practical when direct measurements of distal vascular physiology are unavailable, these strategies rely on generalized downstream assumptions rather than individualized physiological constraints.

This simplification can be problematic even in individuals without diagnosed cerebrovascular disease. Normal cerebral arterial networks show substantial anatomical and functional heterogeneity. Phase-contrast MRI studies in healthy volunteers have demonstrated that cerebral blood flow distribution among the internal carotid, vertebrobasilar, and major intracranial arteries varies with age, sex, and anatomical configuration of the Circle of Willis (CoW) [13]. Thus, even individuals with apparently normal cerebral arterial anatomy may exhibit different regional perfusion patterns depending on collateral anatomy, hemispheric dominance, and distal vascular tone [14,15]. Consequently, boundary strategies that simplify the peripheral vascular bed with a fixed-pressure outlet or assign resistance solely according to diameter-based power laws, including Murray’s law, are insufficient to uniquely determine subject-specific downstream resistance or cerebral blood flow distribution. Fixed-pressure outlets neglect the dominant influence of downstream vascular impedance and therefore produce flow divisions that deviate from in vivo observations [16,17,18]. Murray’s law provides a more structured morphometry-based allocation strategy, but it relies primarily on local branch geometry and cannot account for interindividual variability in distal vascular resistance across heterogeneous perfusion territories. Moreover, when parent–daughter diameter relationships deviate from the assumptions underlying Murray-based scaling, diameter-only resistance allocation may further misrepresent subject-specific flow distribution [17,19]. This limitation is particularly relevant for CFD analyses that aim to resolve hemispheric perfusion asymmetry, collateral flow redistribution, or local biomechanical indices (e.g., WSS) derived from velocity gradients.

Beyond inherent physiological heterogeneity, this limitation becomes even more critical under dynamically altered physiological states that may engage cerebral autoregulatory adaptation. Physiological or interventional conditions such as physical exercise, hypercapnia, orthostatic stress, pharmacological vasodilation, and enhanced external counterpulsation (EECP) can substantially perturb systemic blood pressure and cerebral perfusion [20,21,22]. In response, the cerebrovascular system adjusts distal vascular resistance through cerebral autoregulation (CA) to maintain perfusion homeostasis [23]. However, traditional BC strategies based on fixed outlet pressure or static morphometry-derived resistance allocation are insufficient to represent state-dependent downstream impedance changes.

To achieve a more physiologically constrained representation of the subject-specific cerebrovascular hemodynamic environment, the present study proposes a multimodal data-driven boundary calibration (MDBC) framework for cerebrovascular CFD modeling. By integrating continuous blood pressure (CBP) monitoring and transcranial color-coded Doppler (TCCD) measurements, outlet resistances initially estimated using morphometry-based allocation were iteratively calibrated to match in vivo measured flow waveforms and pressure-derived targets. In the present work, the framework is examined as a proof-of-concept case study in a single healthy volunteer under resting baseline and EECP conditions. EECP was selected as a representative strong hemodynamic perturbation that may engage cerebrovascular autoregulatory adaptation [20,24,25]. The objectives are threefold: (i) to demonstrate the feasibility of incorporating multimodal physiological measurements into outlet boundary calibration; (ii) to compare the resulting MCA velocity waveforms, regional flow allocation, and WSS estimates with those obtained using conventional open-boundary (OB) and static Murray allocation boundary (SMAB) strategies, the latter representing purely morphometry-based resistance allocation; and (iii) to explore whether state-specific calibrated outlet impedances can provide preliminary insight into possible resistance adaptation during EECP. Given the single-subject design, the present study is intended as a preliminary methodological evaluation of technical feasibility rather than an assessment of population-level effectiveness or generalizability. The study establishes an executable workflow for integrating multimodal physiological measurements into subject-specific cerebrovascular CFD modeling, providing a basis for subsequent larger-cohort investigations.

## 2. Materials and Methods

### 2.1. Clinical Materials and Data Acquisition

A 36-year-old healthy male volunteer was recruited for the present study. Magnetic resonance angiography (MRA) was used to reconstruct the three-dimensional (3D) subject-specific cerebrovascular geometry. MRA images were acquired using a 1.5 T MRI scanner (uMR 588, United Imaging Healthcare, Shanghai, China) with a 3D time-of-flight (TOF) sequence. The scan parameters were as follows: repetition time (TR) = 23.6 ms, echo time (TE) = 6.8 ms, flip angle = 20°, and slice thickness = 0.5 mm. The spatial resolution of the reconstructed MR images was 0.43 × 0.43 × 0.50 mm^3^.

In vivo hemodynamic measurements were performed using a TCCD ultrasound system (M9, Mindray, Shenzhen, China) equipped with 2 MHz and 4 MHz phased-array probes. Continuous blood flow velocity waveforms of the bilateral internal carotid arteries (ICAs), vertebral arteries (VAs), and M1 segments of middle cerebral arteries (MCAs) were recorded. Measurements were obtained with the subject in the supine position under resting baseline conditions and during EECP intervention, with a counterpulsation pressure of 30 kPa. Simultaneously, CBP was monitored at the brachial artery for 3 min using a non-invasive CBP monitor (EMS-9D pro, Delica Medical Electronics, Shenzhen, China). Stable representative velocity waveforms were extracted from at least four consecutive cardiac cycles for subsequent inlet prescription and MCA waveform calibration, whereas the 3 min CBP recording was used to derive the pulse pressure (PP) and mean arterial pressure (MAP) targets for global impedance calibration.

### 2.2. Geometry and Meshing

**Figure 1:**
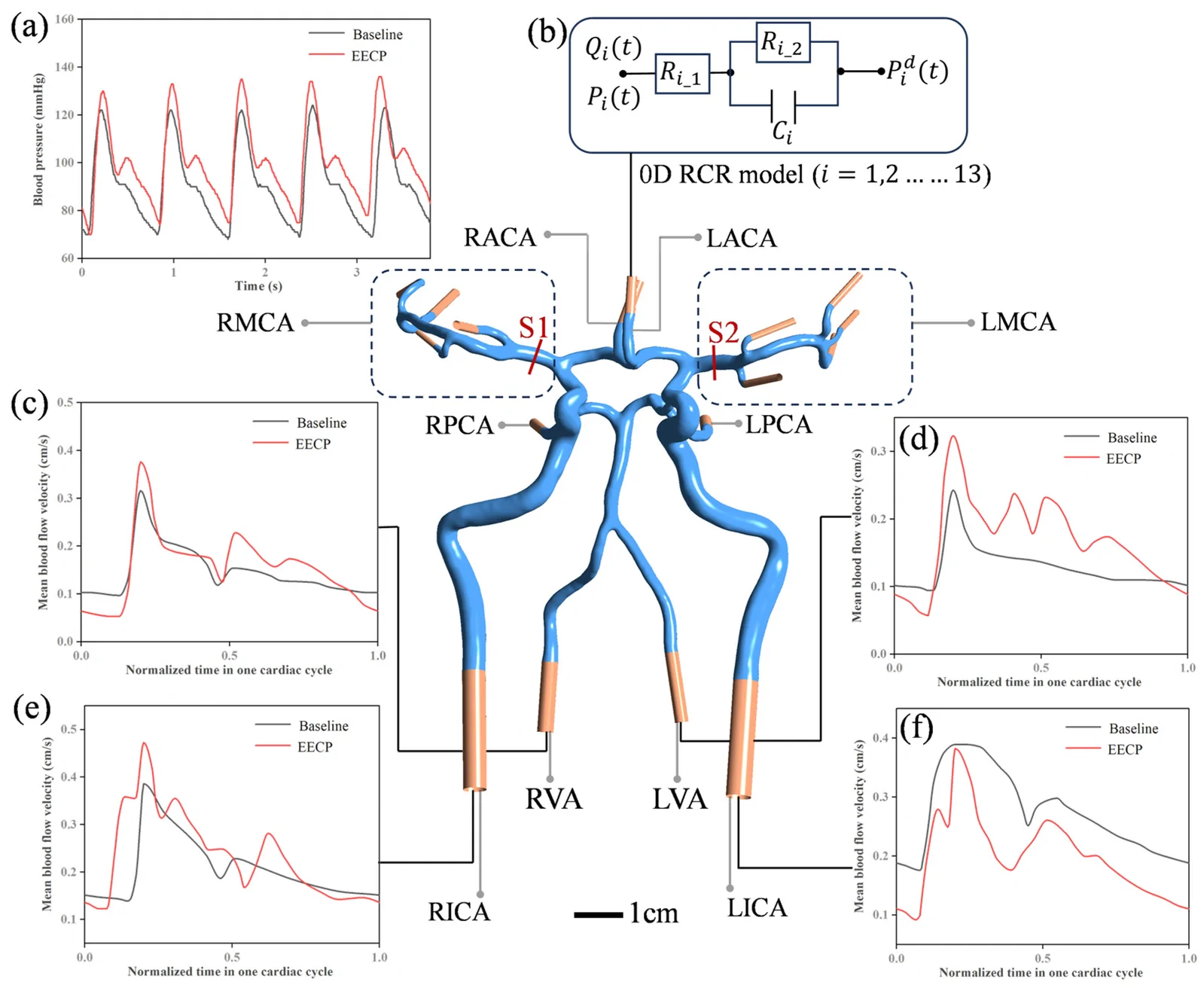
The reconstructed 3D subject-specific CoW geometry with the locations of corresponding inlets and outlets. S1 and S2 indicate the cross-sections at the M1 segments of the bilateral MCAs used for subsequent data extraction. (**a**) CBP waveforms measured at resting baseline and during EECP (representative segment from a 3 min recording). (**b**) The 0D RCR model applied at the outlet boundaries. (**c**–**f**) Time-resolved mean blood flow velocity waveforms measured by TCCD at the four inlets: right vertebral artery (RVA), left vertebral artery (LVA), right internal carotid artery (RICA) and left internal carotid artery (LICA), respectively.

The 3D geometry of the subject-specific CoW arteries was segmented and reconstructed from the acquired MRA data using the open-source software 3D Slicer 5.6.2. As illustrated in Figure 1, the reconstructed fluid domain comprises four inlets (the bilateral ICAs and VAs) and a total of thirteen outlets, including the bilateral anterior cerebral arteries (ACAs), posterior cerebral arteries (PCAs), and nine branches of the MCAs. Notably, the geometric model exhibits an incomplete CoW due to the absence of the right posterior communicating artery (PCoA). Such hypoplasia or absence of communicating arteries is a widespread physiological variant, reported to be present in approximately 34% to 47% of the healthy population [26,27]. Prior to the meshing procedure, the inlet and outlet boundaries were linearly extended by 5–10 times their respective local vessel diameters to ensure that the velocity profiles were fully developed before entering the regions of interest and to reduce non-physical reverse-flow disturbances at the outlets.

An unstructured mesh of the CoW arteries was generated using ICEM (Ansys Inc., Canonsburg, PA, USA), with tetrahedral elements in the main fluid domain and five inflation layers applied near the vessel walls to improve boundary layer resolution. A mesh independence study was performed to determine appropriate mesh quality. Five mesh configurations were tested, comprising approximately 1.61, 3.70, 5.12, 6.86, and 10.10 M elements, with the finest mesh (10.10 M) treated as the reference. TAWSS was calculated and extracted at six arterial cross sections (bilateral MCAs, ACAs, PCAs) under resting baseline and EECP intervention. The root mean square error (RMSE) relative to the reference was calculated. As shown in Table 1, RMSE values were below 6% for meshes about 5.12 M and below 4% for meshes about 6.86 M. Considering the balance between accuracy and computational cost, the 6.86 M mesh was adopted for subsequent simulations.

### 2.3. Solution Methods and Boundary Conditions

Blood was modeled as an incompressible Newtonian fluid with constant density (1060 kg/m^3^) and dynamic viscosity (0.0035 Pa·s). The Newtonian assumption was adopted because the present model primarily comprises the major intracranial arteries of the CoW, with vessel diameters generally ranging from approximately 1.2 to 5 mm, for which this approximation is commonly used in cerebrovascular CFD studies [28,29,30]. Blood flow in the vascular domain was governed by the incompressible Navier–Stokes equations:

(1) $$\left\{\begin{array}{c} \rho \left(\frac{\partial u}{\partial t}+(u\cdot \nabla )u\right)+\nabla p-\mu ∆u=0,\\ \nabla \cdot u=0, \end{array}\right.$$

where u is the velocity vector, p is the pressure, ρ is the fluid density, and μ is the dynamic viscosity.

The vascular model included four inlets and thirteen outlets. Vessel walls were assumed rigid, with a no-slip BC applied. In the absence of subject-specific vascular wall properties, the rigid-wall assumption represents a reasonable modeling simplification and has been widely adopted in cerebrovascular CFD studies [25,30,31,32,33,34]. Time-resolved blood velocity profiles measured by TCCD were imposed at the ICA and VA inlets (Figure 1c–f). For each outlet, we applied a zero-dimensional three-element Windkessel (0D RCR) model as the outlet BC (Figure 1b). For example, the ith outlet consists of a capacitance $C_{i}$, accounting for the compliance of the distal vascular bed, and two resistances $R_{i\_1}$ and $R_{i\_2}$, corresponding to the proximal characteristic impedance and distal peripheral resistance, respectively. The parameters $C_{i}$, $R_{i\_1}$ and $R_{i\_2}$ were outlet-specific and were assigned or calibrated as described in Section 2.4. The 0D RCR model satisfies the following relationship [29]:

(2) $$P_{i}(t)+R_{i\_2}C_{i}\frac{dP_{i}(t)}{dt}=(R_{i\_1}+R_{i\_2})Q_{i}(t)+P_{i}^{d}(t)+R_{i\_1}R_{i\_2}C_{i}\frac{dQ_{i}(t)}{dt}$$

where $P_{i}(t)$ and $Q_{i}(t)$ are the pressure and flow rate at the ith outlet, respectively, and $P_{i}^{d}(t)$ is the downstream vascular pressure. The coupled 3D fluid domain and 0D RCR models were solved using the commercial finite-volume solver ANSYS Fluent 2022 R1 (Ansys Inc., Canonsburg, PA, USA). Dynamic 3D–0D coupling was implemented through customized User-Defined Functions (UDFs), in which the governing differential equations of the RCR models were discretized using a backward time-differencing scheme. Transient simulations were performed with a fixed time-step size of 0.005 s. To reduce the influence of initial transients and ensure that the pulsatile flow reached a periodic state, each simulation was run for three consecutive cardiac cycles. Hemodynamic data used for post-processing and analysis were extracted from the third cardiac cycle. For the CFD solver, the convergence criterion at each time step was defined as normalized residuals below 0.0001 for all governing equations.

### 2.4. Multimodal Data-Driven Boundary Calibration Procedure

**Figure 2:**
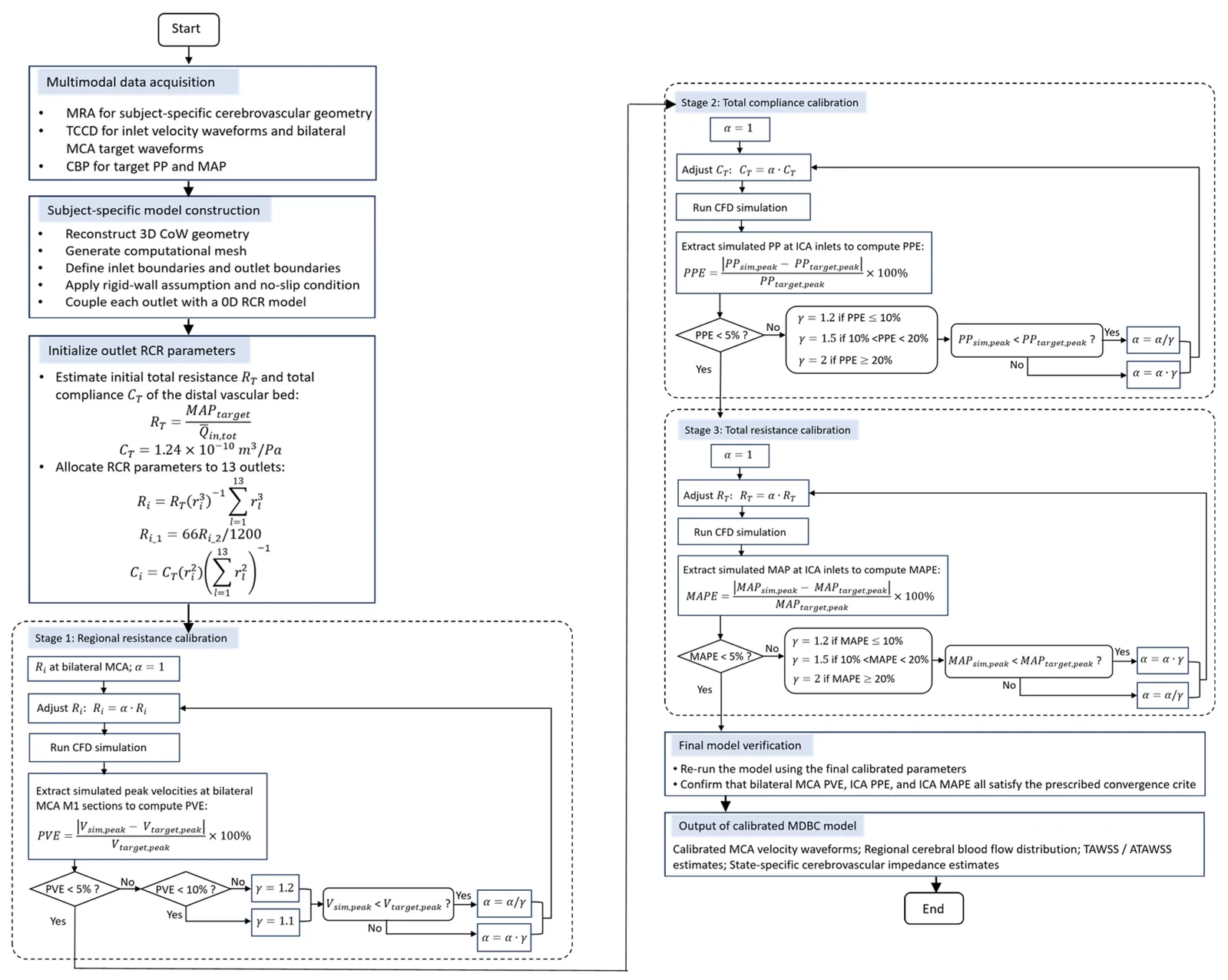
Flowchart of the proposed MDBC algorithm, including multimodal data acquisition, subject-specific model construction, RCR parameter initialization, three-stage calibration of regional resistance, total compliance, and total resistance, and final model verification.

The accuracy of subject-specific cerebrovascular CFD simulations strongly depends on the appropriate assignment of the 0D RCR boundary parameters. In this study, we developed an MDBC framework to personalize outlet resistances and capacitances for each physiological state. Specifically, boundary-parameter calibration was implemented using a heuristic, derivative-free adaptive step-size scaling algorithm. In this algorithm, regional outlet resistance scaling factors, total compliance, and total resistance were sequentially adjusted to match the measured MCA peak velocities, PP, and MAP, respectively. For clarity, a schematic flowchart of the proposed MDBC algorithm has been added to illustrate the complete multi-stage boundary-parameter tuning procedure (Figure 2). The calibration procedure consisted of three progressive stages:

1. The initial total resistance $R_{T}$ and compliance $C_{T}$ were determined before their allocation among the thirteen outlets. The time-averaged total inlet flow, ${\bar{Q}}_{in,tot}$, was calculated as the sum of the mean flow rates measured at the bilateral ICAs and VAs. The initial total resistance was estimated from the 3 min averaged in vivo MAP (${MAP}_{target}$) and the total inlet flow as:
  (3) $$R_{T}=\frac{{MAP}_{target}}{{\bar{Q}}_{in,tot}}$$

The initial total compliance $C_{T}$ was set to $1.24\times 10^{-10}\;m^{3}/Pa$, based on the value reported by Zhou et al. for a cerebral arterial Windkessel model [29]. This literature-derived value was used only as a numerically reasonable initial guess. The initial values of ${\;C}_{i}$, $R_{i\_1}$, and $R_{i\_2}$ in the RCR model were assigned according to the anatomical morphometry of the reconstructed outlet branches. Following the principles of Murray’s law, the terminal branch resistance $R_{i}$ and compliance ${\;C}_{i}$ (i = 1, 2, …, 13) were scaled according to the equivalent radius ($r_{i}$) of the ith outlet utilizing the following relationships [35]:

(4) $$R_{i}={R_{T}(r_{i}^{3})}^{-1}\sum_{l=1}^{13}r_{l}^{3}$$

(5) $$C_{i}=C_{T}(r_{i}^{2}){\left(\sum_{l=1}^{13}r_{l}^{2}\right)}^{-1}$$

where ${\;r}_{l}$ is the equivalent radius of the lth outlet. Once the total branch resistance $R_{i}$ was determined, it was further divided into the characteristic impedance $R_{i\_1}$ and the peripheral resistance $R_{i\_2}$, satisfying $R_{i}\;$=${\;R}_{i\_1}$+$R_{i\_2}$ and the empirically established ratio $R_{i\_1}=66R_{i\_2}$/1200, respectively [36].

2. While Murray’s law provides a common theoretical framework for distributing boundary impedances based on vessel morphometry, it operates on the idealized assumption of minimum energy expenditure across a population-averaged healthy vascular bed [37]. However, significant physiological deviations exist at the individual level, as Murray’s law fails to account for subject-specific anatomical asymmetry, collateral circulation, and autoregulatory adaptations, indicating that morphology alone cannot fully determine the actual downstream vascular impedance. Nevertheless, because direct measurements of distal cerebrovascular resistance are generally unavailable in vivo, Murray-based allocation remains one of the most widely adopted strategies for initializing outlet BCs in cerebrovascular CFD studies [38,39].

To incorporate subject-specific physiological information beyond morphology-based initialization, we implemented a flow-driven fine-tuning strategy centered on the bilateral MCAs. Unlike traditional methods that are constrained by limited outlet information, our framework leverages in vivo TCCD measurements of the bilateral MCAs as physiological calibration targets. Specifically, the initial resistance allocations for the branches of the left middle cerebral artery (LMCA) and right middle cerebral artery (RMCA), derived from Murray’s law, were iteratively adjusted by a regional multiplier, $\alpha_{k}$ (where k denotes the iteration index, initialized at $\alpha_{0}=1.0$). The objective function was defined as the peak velocity error (PVE) at the MCA M1 segments:

(6) $$PVE=\frac{\left|V_{sim,peak}-\;V_{target,peak}\right|}{V_{target,peak}}\times 100\%$$

where $V_{sim,peak}$ and $V_{target,peak}$ were evaluated at the representative M1 cross-sections of the LMCA and RMCA, corresponding to S1 and S2 in Figure 1, respectively. An adaptive adjustment factor, γ, was used to scale $\alpha_{k}$. If $V_{sim,peak}$ < $V_{target,peak}$, the resistance was decreased by updating $\alpha_{k+1}$ = $\alpha_{k}/\gamma$; conversely, if $V_{sim,peak}$ > $V_{target,peak}$, $\alpha_{k+1}$ = $\alpha_{k}\times \gamma$. To accelerate convergence while preventing numerical oscillation, γ was initialized at 1.2 and dynamically reduced to 1.1 once PVE<10%. The iteration for this phase was terminated when the convergence criterion of PVE<5% was satisfied. This feedback-loop calibration was designed to adjust regional outlet resistance allocation so that the simulated bilateral MCA peak velocities more closely matched the subject-specific TCCD measurements.

3. Following the regional flow matching, the global impedance parameters ($R_{T}$ and $C_{T}$) were calibrated to improve agreement between the simulated pressure characteristics and the pressure-derived targets. Firstly, the total compliance $C_{T}$ was tuned to match the simulated PP (${PP}_{sim}$) at the ICA inlets with the in vivo target (${PP}_{target}$), which was extracted as the mean PP from a stable 3 min CBP recording (Figure 1a). The objective function was the relative PP Error (PPE):
  (7) $$PPE=\frac{\left|{PP}_{sim}-\;{PP}_{target}\right|}{{PP}_{target}}\times 100\%$$
  when the ${PP}_{sim}$ was lower than the ${PP}_{target}$, $C_{T}$ was decreased; when the ${PP}_{sim}$ was higher than the ${PP}_{target}$, $C_{T}$ was increased. The adjustment factor was dynamically attenuated based on the residual error: 2.0 (for PPE>20%), 1.5 (for 10%<PPE<20%), and 1.2 (for PPE<10%). Convergence was defined strictly as PPE<5%. Subsequently, the total resistance $R_{T}$ was scaled to align the simulated MAP (${MAP}_{sim}$) with the ${MAP}_{target}$. The objective function was the relative MAP Error (MAPE):
  (8) $$MAPE=\frac{\left|{MAP}_{sim}-\;{MAP}_{target}\right|}{{MAP}_{target}}\times 100\%$$

If the ${MAP}_{sim}$ was lower than the ${MAP}_{target}$, $R_{T}$ was increased; if the ${MAP}_{sim}$ was higher than the target ${MAP}_{target}$, $R_{T}$ was decreased. The same adaptive step-size thresholds used for $C_{T}$ were applied to $R_{T}$, and convergence was defined as MAPE values below 5% at both ICA inlets. During global resistance scaling, the relative outlet resistance distribution determined in the regional calibration step was preserved. It should be noted that the physiological pressure at the ICA inlets is not necessarily identical to brachial arterial pressure because of peripheral pressure amplification and wave-reflection effects [40]. However, direct invasive pressure measurement in the cerebral arteries of healthy volunteers is clinically unfeasible and ethically prohibited. Therefore, brachial artery blood pressure is widely recognized in existing literature as a highly reliable non-invasive surrogate [41,42]. Although brachial arterial pressure may differ from pressure at the ICA inlets, the same non-invasive surrogate was consistently used under both baseline and EECP conditions, thereby reducing the influence of systematic measurement offsets on the within-subject comparison.

Because the regional resistance scaling, total compliance calibration, and total resistance calibration primarily affect MCA peak velocity, PP, and MAP level, respectively, these three calibration stages were performed sequentially. After completion of all stages, the final calibrated model was re-evaluated to confirm that the ICA PPE and ICA MAPE (Table 2) and the MCA PVE (Table 3) all remained within their prescribed convergence thresholds. In the present simulations, each calibration stage converged within approximately 7–8 iterations. Intermediate simulations were terminated once the hemodynamic quantity required for the corresponding calibration stage could be extracted. The complete MDBC calibration required 22 CFD evaluations per physiological state and approximately 12.5 h of wall-clock time using 70 CPU cores, which was considered moderate and manageable for the present offline, subject-specific calibration study.

## 3. Results and Discussion

The present results should be interpreted as a proof-of-concept, within-subject comparison based on a single healthy volunteer, rather than as population-level physiological evidence. The proposed MDBC framework was compared with two conventional outlet BC strategies, namely the OB and SMAB approaches, under resting baseline and EECP conditions. EECP was used as a controlled hemodynamic perturbation that may alter pressure–flow relationships and downstream vascular impedance. First, the simulated bilateral MCA velocity waveforms obtained from the three BC strategies were quantitatively compared with in vivo TCCD measurements to assess waveform fidelity in this subject. We then examined how BC-induced differences propagated into the assessment of regional flow redistribution within the CoW and localized biomechanical metrics, including time-averaged wall shear stress (TAWSS). TAWSS was calculated to quantify the cumulative shear stress over a cardiac cycle, defined as:

(9) $$TAWSS=\frac{1}{T}\int_{0}^{T}\left|\tau_{w}\right|dt$$

where T represents the duration of one cardiac cycle and $\tau_{w}$ denotes the instantaneous WSS vector. Finally, we quantified the calibrated cerebrovascular impedance changes between resting baseline and EECP to explore whether the MDBC framework could provide an indicator of state-specific impedance adaptation potentially associated with CA.

### 3.1. Comparison of Simulated Hemodynamics with In Vivo Measurements

**Figure 3:**
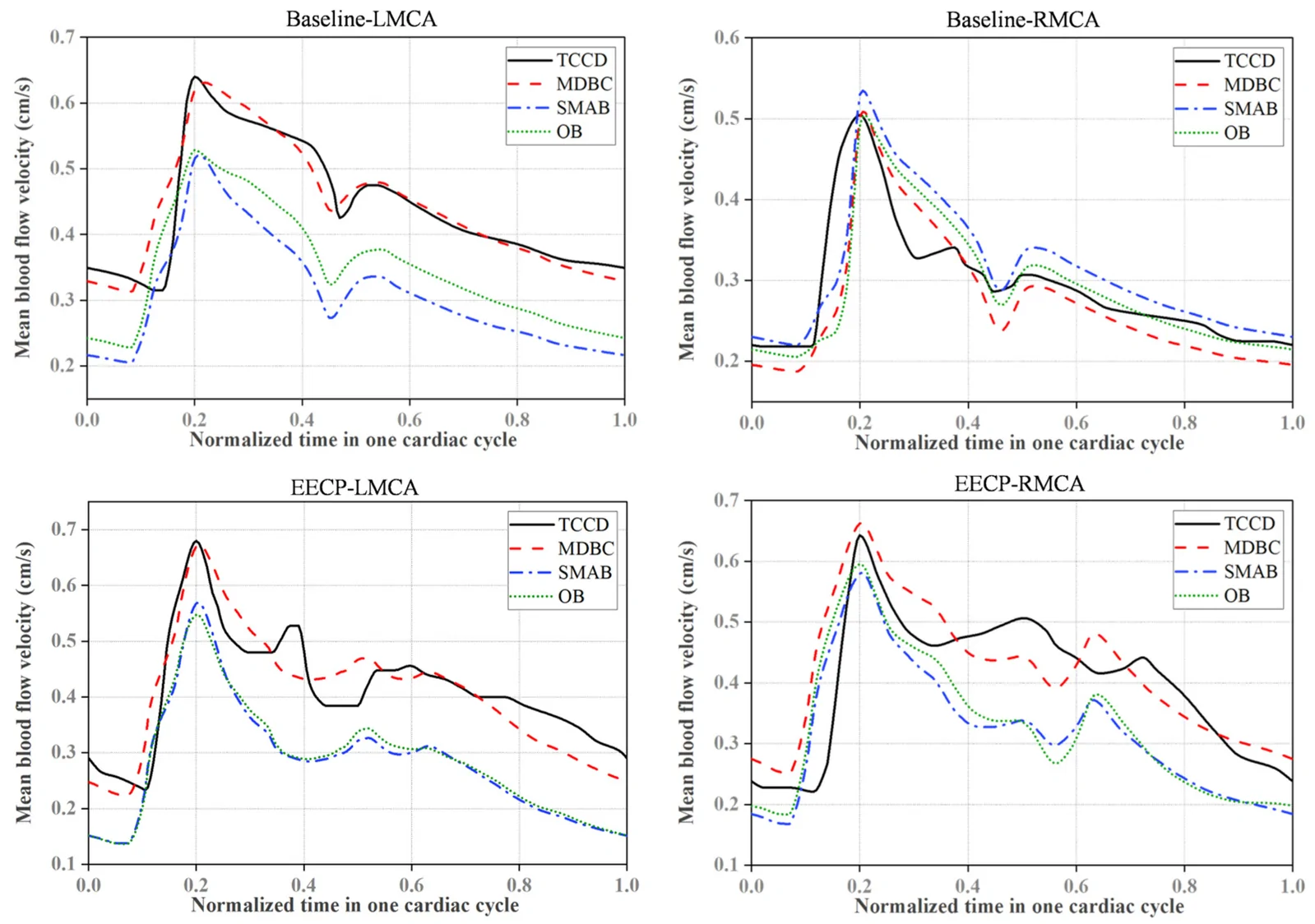
Comparison of simulated velocity waveforms (MDBC, SMAB, and OB) with in vivo TCCD measurements at the bilateral MCAs.

Representative waveform comparisons between the simulations and in vivo TCCD measurements are shown in Figure 3, with quantitative error metrics summarized in Table 3. The simulated flow velocity waveforms were extracted from the representative M1 cross-sections of the bilateral MCAs, corresponding to S1 and S2 in Figure 1. Given that the subject had uniform M1 segments without apparent stenosis, cross-sectional mean velocity was used to quantify post-calibration agreement between the simulated and measured MCA waveforms. Relative root mean square error (rRMSE) was calculated to evaluate the overall waveform agreement throughout the cardiac cycle, whereas PVE and time-averaged velocity error (TAVE) were used to characterize the matching of systolic transient responses and cycle-averaged cerebral perfusion, respectively.

**Table 3:** Error analysis of simulated blood flow velocities across different BC frameworks under baseline and EECP states.

Physiological State	Artery Branch	Metric	OB	SMAB	MDBC
Baseline	LMCA	rRMSE	22.85%	30.89%	7.19%
		PVE	17.39%	18.64%	1.41%
		TAVE	20.87%	29.56%	0.67%
Baseline	RMCA	rRMSE	16.76%	15.67%	15.44%
		PVE	0.05%	5.98%	0.82%
		TAVE	0.73%	6.92%	6.88%
EECP	LMCA	rRMSE	32.59%	33.47%	11.24%
		PVE	19.47%	16.20%	0.90%
		TAVE	30.64%	31.47%	1.42%
EECP	RMCA	rRMSE	27.62%	27.35%	17.45%
		PVE	7.38%	9.55%	3.20%
		TAVE	17.94%	20.17%	4.53%

The MDBC framework showed closer agreement with the measured MCA velocity waveforms than the OB and SMAB strategies under both resting and EECP conditions. At resting baseline, the MDBC method reduced the rRMSE for the LMCA to 7.19%, compared with 22.85% for OB and 30.89% for SMAB, while maintaining low PVE (1.41%) and TAVE (0.67%). These results indicate that, in this subject, a constant-pressure outlet or morphometry-only resistance allocation did not adequately reproduce the measured LMCA waveform, even under resting conditions. Although the OB method incidentally exhibited low PVE and TAVE for the RMCA at baseline, the MDBC method maintained competitive accuracy and achieved the lowest rRMSE (15.44%) among the three strategies, suggesting better agreement with the temporal morphology of the pulsatile velocity waveform rather than only with isolated peak or cycle-averaged values.

During EECP, the advantages of the MDBC framework became particularly pronounced. For the LMCA, OB and SMAB yielded rRMSE values above 32% and TAVE values above 30%, whereas MDBC reduced the rRMSE to 11.24% and the TAVE to 1.42%. For the RMCA, MDBC also produced lower rRMSE, PVE, and TAVE values than the two conventional approaches. These results suggest that state-specific calibration using multimodal physiological measurements improved the agreement between simulated and measured MCA velocities in this pressure-perturbed condition.

Taken together, these findings indicate that, in this healthy volunteer, the proposed MDBC framework achieved closer agreement with the measured MCA velocity waveforms than the conventional BC strategies in this subject. In contrast, conventional BC strategies exhibited non-negligible deviations from in vivo measurements under both resting and EECP conditions. These discrepancies should not be regarded merely as local waveform errors at the MCA segments. Because the CoW is a hydraulically coupled vascular network, inaccurate resolution of bilateral MCA velocity waveforms may propagate into the estimation of interhemispheric perfusion balance, regional flow redistribution, and localized biomechanical indices such as WSS, leading to misinterpretation of the hemodynamic effects of intervention. Therefore, in this proof-of-concept case, incorporating multimodal physiological measurements into boundary calibration reduced the observed simulation–measurement discrepancies and may be important for improving the physiological plausibility of subject-specific cerebrovascular CFD analyses. Whether similar benefits occur across larger healthy cohorts or in patients with cerebrovascular disease, where stenosis, infarcts, collateral compensation, and impaired autoregulation may further alter downstream impedance [43,44,45,46], remains to be evaluated in future studies.

### 3.2. Boundary Condition Effects on Regional Flow Redistribution

**Figure 4:**
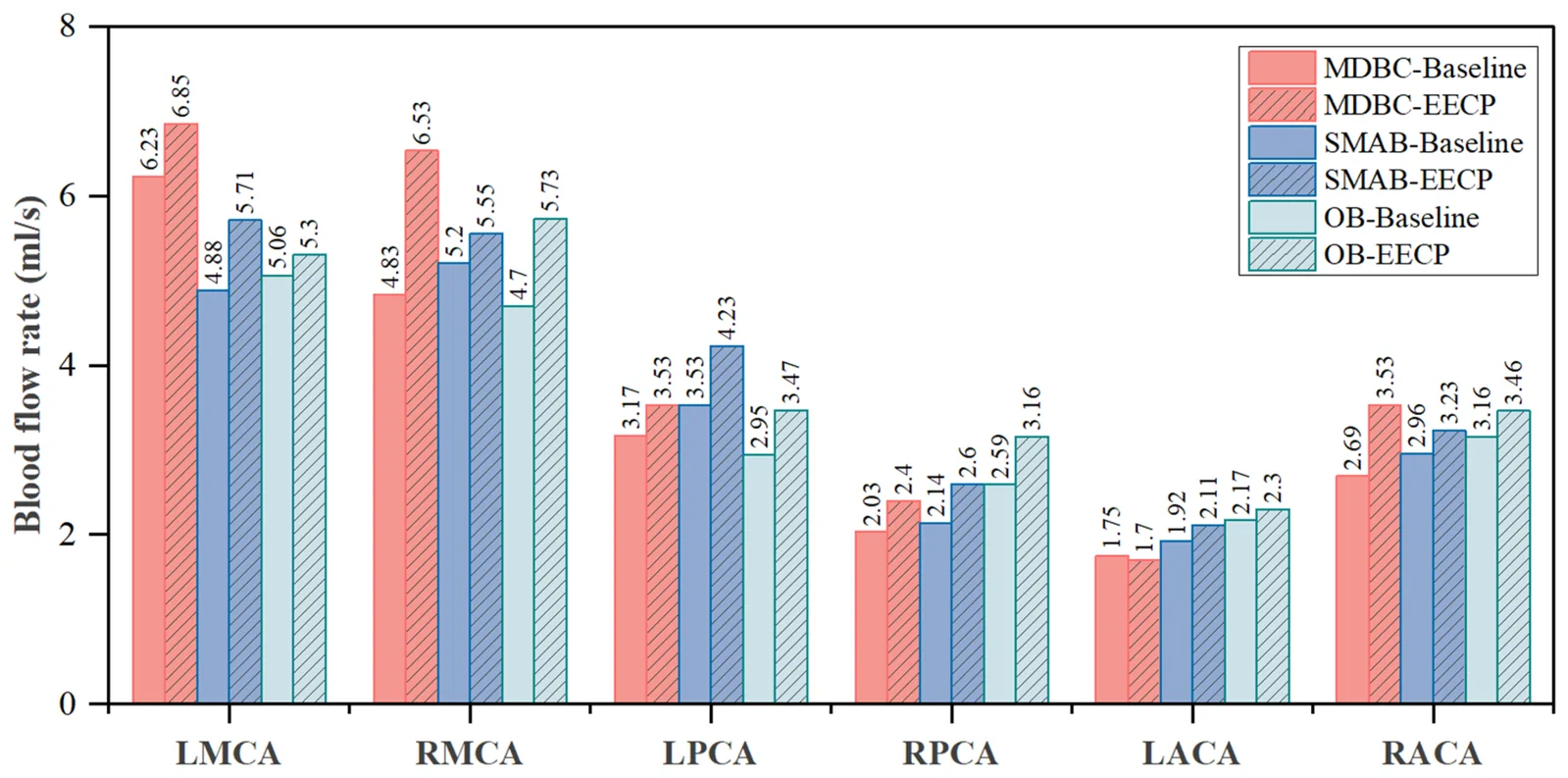
Comparison of regional cerebral blood flow distribution across major branches of the CoW under different BC strategies (OB, SMAB, and MDBC) at resting baseline and during EECP intervention.

To examine how BC-induced waveform discrepancies propagated into broader hemodynamic assessments, regional flow distribution within the CoW network was compared among the three modeling strategies. As illustrated in Figure 4, clear inter-method differences were observed not only in baseline flow allocation but also in the predicted redistribution of cerebral perfusion during EECP.

At resting baseline, MDBC yielded a left-dominant MCA flow pattern that was consistent with the bilateral MCA velocity asymmetry incorporated into the calibration, with flow rates of 6.23 mL/s and 4.83 mL/s in the LMCA and RMCA, respectively. In contrast, the SMAB method predicted a more symmetric MCA distribution (4.88 mL/s vs. 5.20 mL/s), and the OB method produced a smaller left–right difference (5.06 mL/s vs. 4.70 mL/s). Given the waveform comparison against TCCD measurements in Section 3.1, these results suggest that the conventional outlet BC strategies tended to homogenize the bilateral MCA perfusion pattern in this case, whereas MDBC preserved the interhemispheric asymmetry implied by the measured velocity data.

During EECP, the differences among the three strategies became more pronounced. Both SMAB and OB predicted generalized increases in absolute flow across all analyzed vascular territories. However, analysis of relative branch contributions showed that these conventional strategies largely preserved a nearly fixed outlet allocation pattern between resting baseline and EECP. In particular, both methods maintained an approximately symmetric hemispheric flow distribution between the left and right territories under both conditions, with most individual branch allocation changes remaining within approximately 1.5 percentage points. For example, SMAB estimated only a modest 6.7% increase in RMCA absolute flow, accompanied by minimal redistribution across the CoW. These results suggest that static outlet strategies primarily responded to augmented inlet flow by proportionally scaling downstream perfusion, rather than resolving substantial state-dependent redistribution within the CoW. In contrast, the MDBC framework predicted a more heterogeneous redistribution pattern during EECP. The total flow allocated to the left and right hemispheres shifted from 53.8% vs. 46.2% at baseline to 49.33% vs. 50.67% during EECP. Consistent with this global shift, the relative contribution of the RMCA increased from 23.35% to 26.59%, whereas the shares of the LMCA and LACA decreased from 30.08% to 27.91% and from 8.44% to 6.93%, respectively. These allocation changes were accompanied by spatially heterogeneous absolute flow responses, including a 35.2% increase in RMCA flow from 4.83 to 6.53 mL/s and a slight reduction in LACA flow from 1.75 to 1.70 mL/s.

Physiologically, the CoW is thought to facilitate redistribution of cerebral blood supply under altered perfusion conditions rather than simply passively transmitting increased systemic inflow [47,48,49]. Therefore, accurately resolving such spatially non-uniform redistribution is considerably more informative than reproducing global flow augmentation alone. In this proof-of-concept case, MDBC was constrained by subject-specific MCA velocity measurements and therefore yielded a regional flow distribution that was more consistent with the available in vivo information than the distributions obtained using the conventional boundary strategies. However, because independent flow measurements were not available for the ACA and PCA territories, the physiological accuracy of the predicted whole-network redistribution cannot be established from the present single-subject data. This study does not establish the general redistribution behavior of EECP across subjects, but it demonstrates that, at least in this individual case, BC choice substantially affected the predicted regional perfusion response.

### 3.3. Boundary Condition Effects on Localized Biomechanical Indices

Beyond macroscopic flow redistribution, the choice of outlet BCs also substantially affected the estimated localized biomechanical environment within the bilateral MCAs. The MCAs were selected for detailed analysis because they are major cerebral perfusion vessels and are frequently involved in cerebrovascular disease [50]. To quantify localized hemodynamic loading, two key metrics were introduced: area-averaged TAWSS (ATAWSS) and the proportion of high-risk vascular areas. The latter was defined as regions exposed to abnormally low (<1 Pa) or excessively high (>10 Pa) TAWSS, both of which are strongly associated with endothelial dysfunction, atherosclerosis progression, and pathological vascular remodeling [51,52].

**Figure 5:**
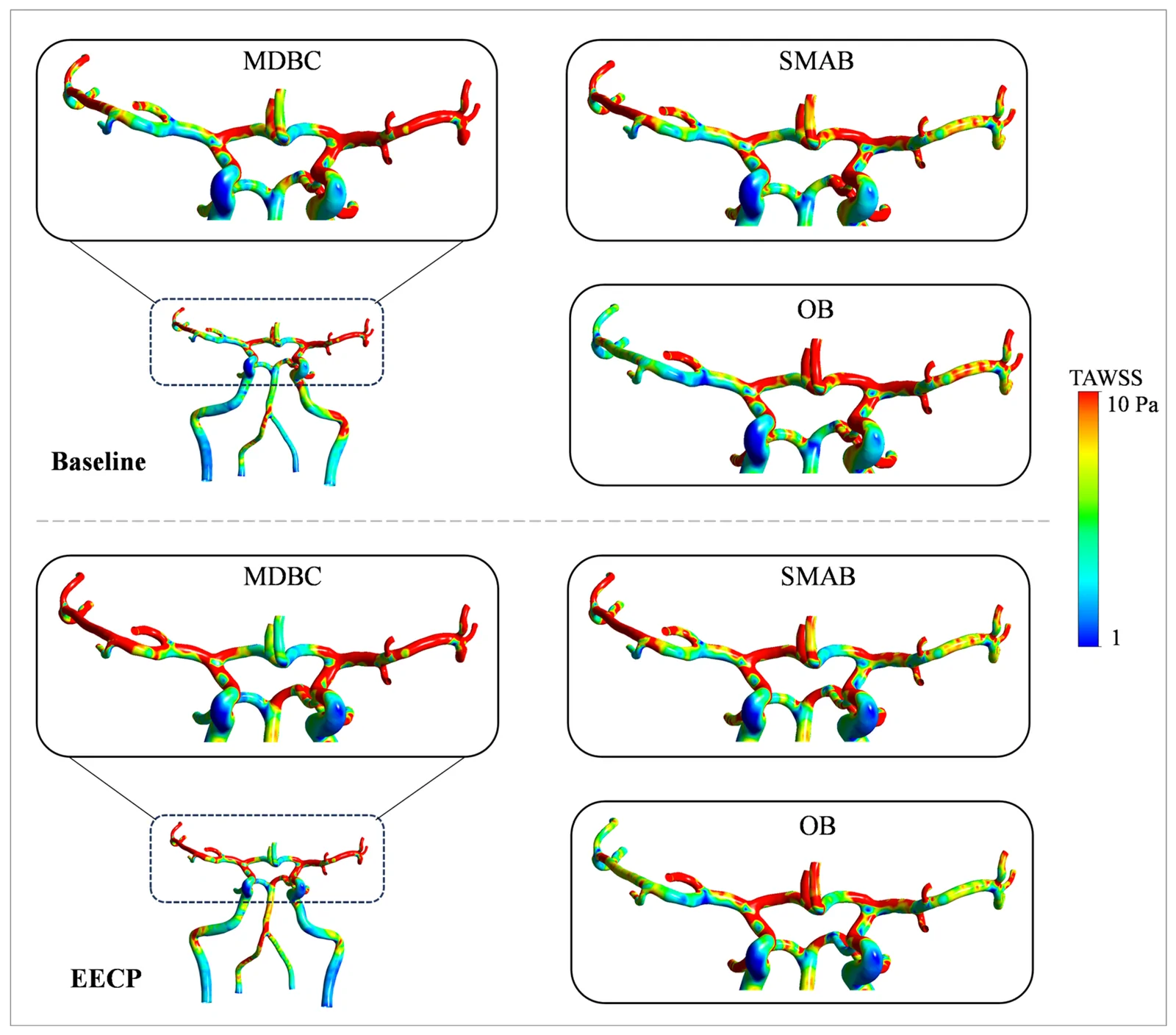
Spatial distribution of TAWSS calculated by OB, SMAB, and MDBC frameworks at resting baseline and during EECP intervention.

**Figure 6:**
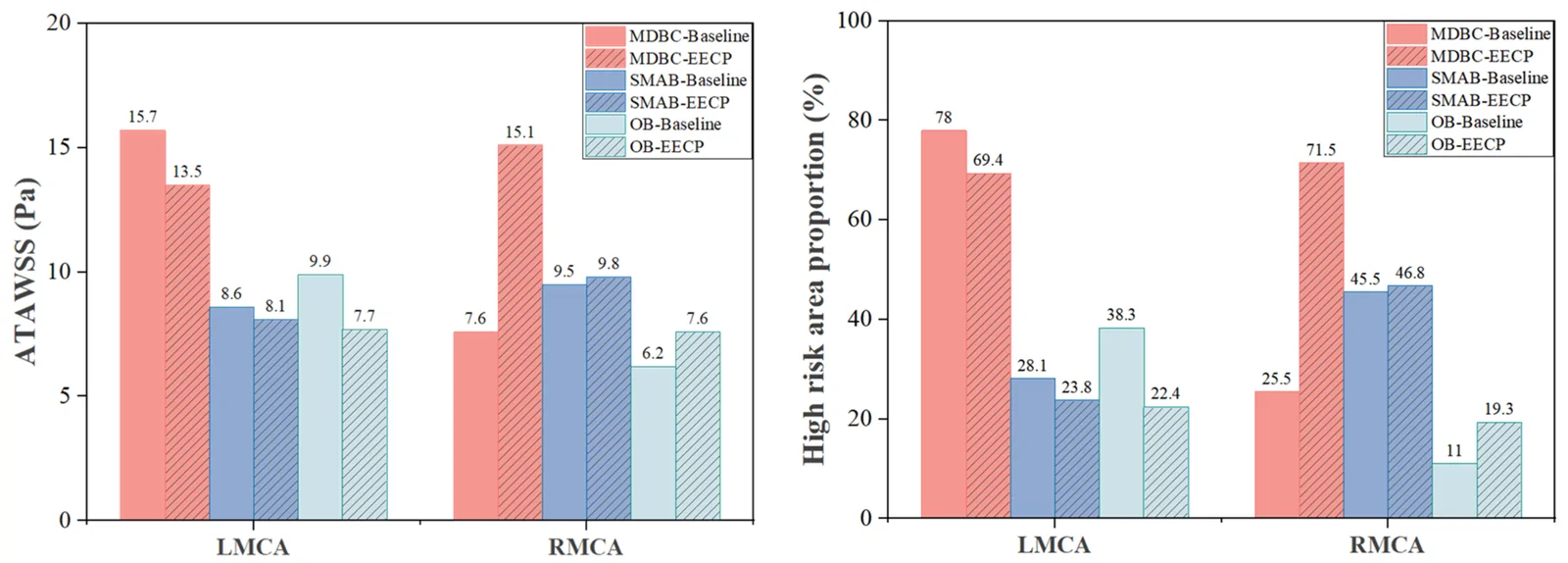
Variations in ATAWSS magnitude (**left**) and percentage of high-risk areas (**right**) within the LMCA and RMCA, calculated by OB, SMAB, and MDBC models at resting baseline and during EECP intervention.

As shown in Figure 5 and Figure 6, the three BC strategies produced markedly different local hemodynamic estimates under the same physiological state. At resting baseline, the MDBC framework predicted an ATAWSS of 15.66 Pa for the LMCA, with a corresponding high-risk area proportion of 78.03%. Conversely, these values were substantially underestimated by the SMAB (ATAWSS: 8.56 Pa; High-risk area: 28.13%) and OB (ATAWSS: 9.87 Pa; High-risk area: 38.27%) methods. MDBC also predicted a clear bilateral difference, with the LMCA ATAWSS nearly twice that of the RMCA (15.66 Pa vs. 7.65 Pa). By contrast, the conventional BC strategies yielded more homogenized bilateral TAWSS distributions. Because TAWSS is derived from near-wall velocity gradients, these discrepancies are consistent with the MCA waveform differences reported in Section 3.1 and indicate that outlet BC selection can substantially influence localized biomechanical estimates in this subject.

During EECP, inter-method differences in localized biomechanical indices became more evident. Under MDBC, RMCA ATAWSS increased from 7.65 Pa at baseline to 15.11 Pa during EECP, accompanied by an increase in the high-risk area proportion from 25.46% to 71.50%. In contrast, the conventional strategies predicted much smaller changes. For example, SMAB predicted only a slight increase in RMCA ATAWSS from 9.50 Pa to 9.75 Pa, with the corresponding high-risk area proportion remaining nearly unchanged (45.54% to 46.82%). These results suggest that, in this proof-of-concept case, localized biomechanical responses to EECP were highly dependent on the predicted regional flow redistribution and therefore sensitive to outlet BC assignment.

Overall, the comparison indicates that simplified outlet BC strategies may mask localized hemodynamic changes when their predicted flow waveforms deviate from in vivo measurements. By constraining the simulation with measured MCA velocities and pressure-derived targets, MDBC provided biomechanical estimates that were more consistent with the subject-specific flow information available in this study. This capability is essential for more accurately evaluating how interventions (e.g., EECP) alter the individualized hemodynamic environment within the CoW. However, these findings do not establish general WSS responses to EECP; rather, they support the methodological value of multimodal boundary calibration for reducing downstream uncertainty in localized hemodynamic assessment. Future studies are required to determine whether this advantage is preserved across larger cohorts and patient populations.

### 3.4. Estimated State-Specific Cerebrovascular Impedance Changes During EECP

**Figure 7:**
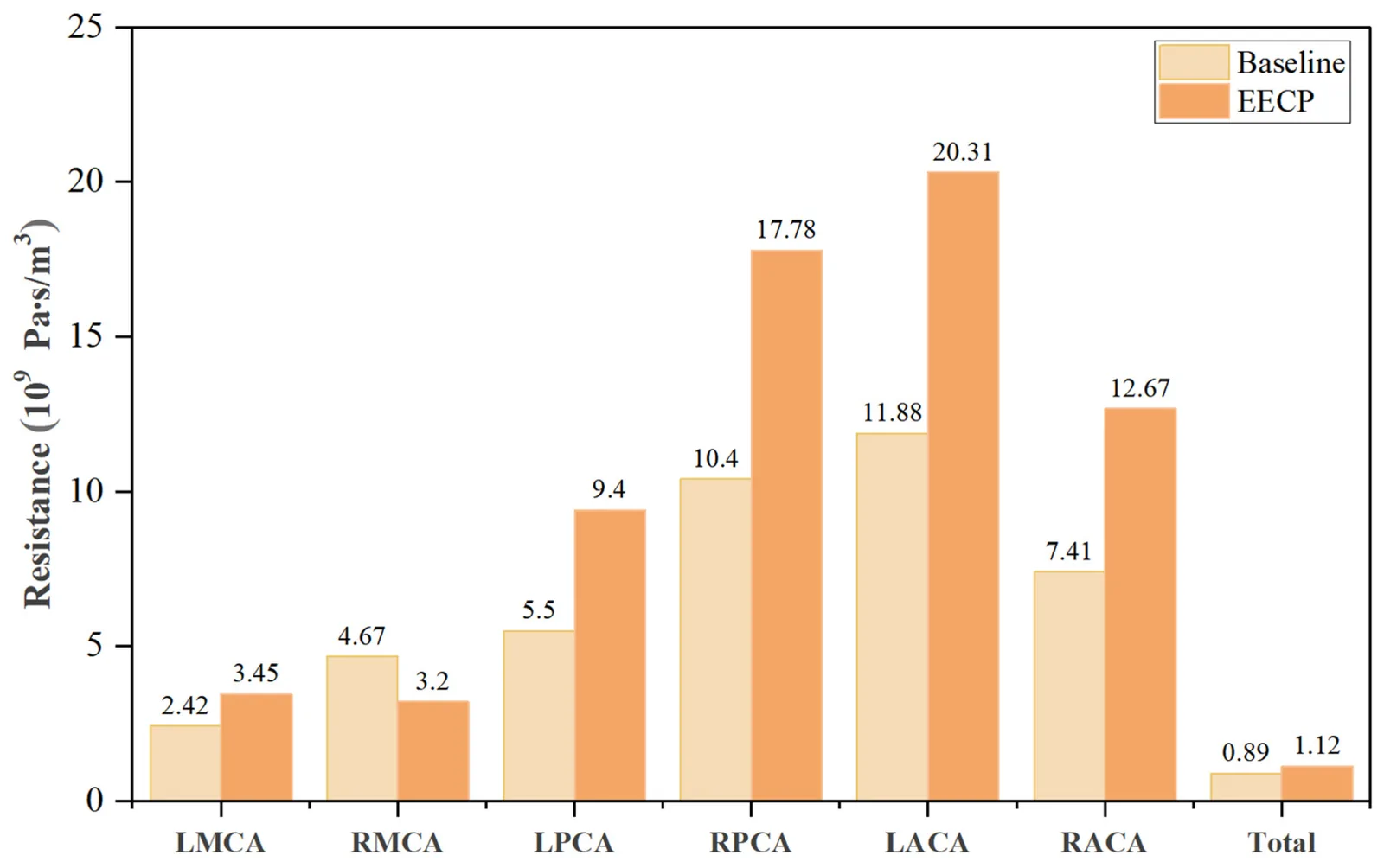
Comparison of regional outlet resistances in different cerebral vascular territories and total resistance between baseline and EECP conditions.

In addition to improving agreement with measured flow waveforms and altering downstream estimates of regional flow and WSS, the MDBC framework provided a means of estimating state-specific cerebrovascular impedance changes from multimodal physiological measurements. By integrating TCCD-derived flow information and CBP monitoring into the boundary calibration process, state-specific outlet impedance distributions were calibrated under resting baseline and EECP conditions. The resulting calibrated parameters suggested substantial changes in cerebrovascular resistance during EECP in this subject (Figure 7). At the global level, the total cerebrovascular resistance increased by 25.8% during EECP, rising from $8.9\times 10^{8}$ to $1.12\times 10^{9}\;Pa\cdot s/m^{3}$. Because EECP can augment systemic venous return and elevate MAP through diastolic counterpulsation [25], an increase in downstream cerebrovascular resistance is physiologically consistent with autoregulatory buffering against pressure-induced hyperperfusion [53]. However, because this inference is derived from a single subject and from calibrated model parameters rather than direct vascular reactivity measurements, it should be interpreted as a model-derived indicator of possible CA-related impedance adaptation rather than direct proof of CA.

Region-specific impedance estimates further suggested spatial heterogeneity in the response to EECP. The resistances assigned to the bilateral ACA and PCA territories increased by approximately 70.9%, and the LMCA resistance increased by 42.4%, from 2.42 to 3.45 $\times \;10^{9}\;Pa\cdot s/m^{3}$. Conversely, the RMCA showed a decrease in calibrated resistance by 31.6%, from 4.67 to 3.20$\;\times 10^{9}\;Pa\cdot s/m^{3}$. Although limited to one individual, these model-derived estimates suggest that the impedance response during EECP may not be spatially uniform across the cerebral network. This potential regional heterogeneity aligns with previous physiological studies indicating that CA efficiency and sensitivity can differ significantly among distinct vascular territories (e.g., between the anterior and posterior circulations) [54].

These preliminary findings illustrate the potential utility of the MDBC framework for inferring individualized, state-specific cerebrovascular impedance changes from non-invasive multimodal measurements. This capability may be useful for future studies investigating why cerebral blood-flow responses to EECP differ among individuals and patient groups. Previous studies have reported heterogeneous cerebral flow augmentation during EECP in healthy subjects and patients with ischemic cerebrovascular disease [55,56], but the mechanisms underlying this variability remain incompletely understood. One possible explanation is that interindividual differences in autoregulatory capacity may modulate the cerebral flow response to EECP: individuals with relatively preserved autoregulation may increase downstream resistance to buffer pressure-induced perfusion changes, whereas patients with impaired autoregulation may exhibit more passive pressure-dependent flow augmentation. By enabling estimation of state-specific impedance adaptation, the MDBC framework may provide a computational tool for testing this hypothesis in future cohort studies and for further clarifying the hemodynamic mechanisms of EECP.

## 4. Limitations

Some limitations should be acknowledged. First, the present study included only a single healthy volunteer because its primary aim was to provide a proof-of-concept evaluation of whether the MDBC framework is feasible under two distinct physiological states. Accordingly, no statistical inference regarding the performance of MDBC relative to conventional BC strategies can be drawn from the present study. Moreover, the adjustment factors, adaptive step sizes, and convergence thresholds used in the present study were prespecified as pragmatic numerical settings for this proof-of-concept implementation. Their robustness and transferability across individuals with different anatomical and physiological characteristics remain to be systematically evaluated in larger cohorts and through sensitivity analyses. Further testing in larger cohorts is required to determine the generalizability and robustness of the proposed workflow.

Second, the feature-based calibration using PVE, PPE, and MAPE prioritizes agreement in regional peak flow, pressure pulsatility, and mean pressure level, respectively, but does not fully constrain the complete velocity and pressure waveforms throughout the cardiac cycle. Future studies should investigate full-waveform or multi-objective calibration and quantify the resulting parameter and hemodynamic uncertainties.

The simplified assumptions of rigid vessel walls and Newtonian blood rheology may affect the estimated magnitudes of certain hemodynamic parameters. Future studies should further evaluate the proposed boundary calibration framework using non-Newtonian blood rheology and compliant-wall models to determine whether the feasibility and relative performance of the MDBC approach are preserved under more physiologically detailed modeling conditions.

## 5. Conclusions

This study developed a multimodal data-driven boundary calibration (MDBC) framework for subject-specific cerebrovascular CFD modeling by integrating TCCD-derived velocity measurements and continuous blood-pressure monitoring into outlet RCR parameter calibration. In a single healthy volunteer evaluated under resting baseline and EECP conditions, MDBC achieved closer agreement with in vivo MCA velocity waveforms than conventional open-boundary and static Murray allocation boundary strategies. In this case, improved waveform agreement was accompanied by substantially different estimates of regional flow redistribution and localized WSS-related indices compared with conventional boundary-condition strategies. Our findings indicate that generic outlet boundary assumptions may introduce non-negligible errors in subject-specific cerebrovascular CFD simulations, whereas multimodal physiological calibration can provide a more physiologically constrained representation of the individual hemodynamic state.

Moreover, the calibrated MDBC parameters provided model-derived estimates of state-specific cerebrovascular impedance changes during EECP, which may reflect CA-related regulation triggered by pressure perturbation and offer a new perspective for investigating interindividual variability in cerebral hemodynamic responses to EECP. Although the single-subject design precludes population-level conclusions or claims regarding the general hemodynamic effects of EECP, this proof-of-concept study establishes a clearly specified and executable methodological workflow. Future validation in larger healthy cohorts and patients with cerebrovascular disease is needed to assess the robustness, generalizability, and clinical relevance of the MDBC framework. With further validation, this approach may support more physiologically constrained patient-specific hemodynamic assessment and facilitate future investigations of individualized cerebrovascular responses to interventions such as EECP.

## Figures and Tables

**Table 1:** Mesh-independence analysis based on TAWSS RMSE relative to the finest mesh.

		Baseline				EECP			
Mesh quantity		1.61 M	3.70 M	5.12 M	6.86 M	1.61 M	3.70 M	5.12 M	6.86 M
RMSE	ACAs	10.82%	7.49%	5.47%	3.40%	11.39%	7.61%	5.42%	3.31%
	MCAs	18.60%	14.62%	5.59%	2.32%	18.42%	14.60%	5.55%	2.24%
	PCAs	8.44%	7.40%	5.57%	2.64%	8.41%	7.33%	5.56%	2.74%

**Table 2:** Final calibration errors for PP and MAP at the ICA inlets.

Physiological State	ICA Inlet	${PP}_{sim}$ (mmHg)	${MAP}_{sim}$ (mmHg)	${PP}_{target}$ (mmHg)	${MAP}_{target}$ (mmHg)	PPE(%)	MAPE(%)
Baseline	LICA	51.98	88.71	53.83	87.11	3.43	1.84
	RICA	52.33	91.10			2.79	4.58
EECP	LICA	55.26	99.42	55.40	95.52	0.25	4.08
	RICA	52.84	95.90			4.62	0.40

## Data Availability

The data presented in this study are available upon request from the corresponding author.

## Abbreviations

The following abbreviations are used in this manuscript:

0D	zero-dimensional
3D	three-dimensional
ACA	anterior cerebral artery
ATAWSS	area-averaged time-averaged wall shear stress
BC	boundary condition
CA	cerebral autoregulation
CBP	continuous blood pressure
CFD	computational fluid dynamics
CoW	Circle of Willis
EECP	enhanced external counterpulsation
FSI	fluid–structure interaction
ICA	internal carotid artery
LACA	left anterior cerebral artery
LICA	left internal carotid artery
LMCA	left middle cerebral artery
LPCA	left posterior cerebral artery
LVA	left vertebral artery
MAP	mean arterial pressure
MAPE	relative mean arterial pressure error
MCA	middle cerebral artery
MDBC	multimodal data-driven boundary calibration
MRA	magnetic resonance angiography
MRI	magnetic resonance imaging
OB	open boundary
PCA	posterior cerebral artery
PCoA	posterior communicating artery
PP	pulse pressure
PPE	relative pulse pressure error
PVE	peak velocity error
RACA	right anterior cerebral artery
RCR	three-element Windkessel
RICA	right internal carotid artery
RMCA	right middle cerebral artery
RPCA	right posterior cerebral artery
rRMSE	relative root mean square error
RMSE	root mean square error
RVA	right vertebral artery
SMAB	static Murray allocation boundary
TAVE	time-averaged velocity error
TAWSS	time-averaged wall shear stress
TCCD	transcranial color-coded Doppler
TE	echo time
TOF	time-of-flight
TR	repetition time
UDF	user-defined function
VA	vertebral artery
WSS	wall shear stress
